# A feasibility study of reduced full-of-view synthetic high-*b*-value diffusion-weighted imaging in uterine tumors

**DOI:** 10.1186/s13244-022-01350-0

**Published:** 2023-01-16

**Authors:** Qian Tang, Qiqi Zhou, Wen Chen, Ling Sang, Yu Xing, Chao Liu, Kejun Wang, Weiyin Vivian Liu, Lin Xu

**Affiliations:** 1 Department of Radiology, Taihe Hospital, Hubei University of Medicine, Shiyan, Hubei China; 2 Biomedical Engineering College, Taihe Hospital, Hubei University of Medicine, Shiyan, Hubei China; 3 GE Healthcare, Beijing, China

**Keywords:** Cervical cancer, Reduced field-of-view diffusion-weighted imaging, Synthetic diffusion-weighted image, Magnetic resonance image

## Abstract

**Objectives:**

This study aimed to evaluate the feasibility of reduced full-of-view synthetic high-*b* value diffusion-weighted images (rFOV-syDWIs) in the clinical application of cervical cancer based on image quality and diagnostic efficacy.

**Methods:**

We retrospectively evaluated the data of 35 patients with cervical cancer and 35 healthy volunteers from May to November 2021. All patients and volunteers underwent rFOV-DWI scans, including a 13b-protocol: *b* = 0, 25, 50, 75, 100, 150, 200, 400, 600, 800, 1000, 1200, and 1500 s/mm^2^ and a 5b-protocol: *b* = 0, 100, 400, 800,1500 s/mm^2^. rFOV-syDWIs with *b* values of 1200 (rFOV-syDWI_b=1200_) and 1500 (rFOV-syDWI_b=1500_) were generated from two different multiple-b-value image datasets using a mono-exponential fitting algorithm. According to homoscedasticity and normality assessed by the Levene’s test and Shapiro–Wilk test, the inter-modality differences of quantitative measurements were, respectively, examined by Wilcoxon signed-rank test or paired t test and the inter-group differences of ADC values were examined by independent t test or Mann–Whitney U test.

**Results:**

A higher inter-reader agreement between SNRs and CNRs was found in 13b-protocol and 5b-protocol rFOV-syDWI_b=1200/1500_ compared to 13b-protocol rFOV-sDWI_b=1200/1500_ (*p* < 0.05). AUC of 5b-protocol syADC_mean,b=1200/1500_ and syADC_minimum,b=1200/1500_ was equal or higher than that of 13b-protocol sADC_mean,b=1200/1500_ and sADC_minimum,b=1200/1500_.

**Conclusions:**

rFOV-syDWIs provide better lesion clarity and higher image quality than rFOV-sDWIs. 5b-protocol rFOV-syDWIs shorten scan time, and synthetic ADCs offer reliable diagnosis value as scanned 13b-protocol DWIs.

## Key points

- rFOV-syDWI provided better lesion clarity and image quality while shortening scan time.
- Scanned and synthetic ADCs have the same diagnostic value for cervical cancer.
- Scanned and synthetic ADC_mean, minimum_ can distinguish cervical lesions from normal tissue

## Introduction

Diffusion-weighted imaging (DWI) is an essential functional imaging technology for noninvasively detecting diffusion movement orientation and local restriction degree of water molecules in living tissues, thus indirectly reflecting the changes in tissue microstructure [1]. It can provide both good visual clarity of tumors with high signal intensity and quantitative information like apparent diffusion coefficient (ADC), so a widely used single-shot echo-planar imaging (EPI) DWI is applied to the diagnosis and treatment of various diseases including gynecological tumors [2]. Previous studies have shown that ADC value can be used to differentiate cervical cancer from normal cervix tissues and predict the stage and type of cervical cancer [3, 4]. DWI can also effectively assess the invasion depth of the uterine corpus and infiltration range of parauterine organ by cervical cancer. Therefore, DWI is of importance for the evaluation and treatment of uterine tumors.

Currently, the most commonly used diffusion-weighted imaging (DWI) is used in either clinical diagnosis or scientific research of uterine diseases using single-shot k-space trajectory echo-planar imaging (SS-EPI). However, SS-EPI DWI is prone to have image distortion, blurring, and signal loss due to its narrow bandwidth in the phase coding direction and long readout time [5, 6]. In recent years, DWI with reduced field-of-view (r-FOV) in the phase-encoding direction has been developed to overcome magnetic susceptibility and motion artifacts [7] that occur in full field-of-view (f-FOV) acquisition including signals from liquid, gas, and other substances other than target tissues [7, 8]. rFOV-DWI reduces the number of phase-encoding lines and readout time via selective RF excitation pulse in collocation with frequency encoding gradient and increases echo signal intensity using the 180° refocusing pulse [9, 10], eventually improving image quality with higher image resolution, less magnetic sensitivity and motion artifact. Up to date, it has been in particular applied to spinal cord imaging, pancreatic lesions, and cervical cancer [7, 11, 12].

Another challenge of DWI applications in human body is the selection of diffusion sensitive factors (*b* values). Currently, *b* values used for disease evaluation in most in vivo DWI studies usually range between 0 and 1000 s/mm^2^ [13], and *b* values greater than 1000 s/mm^2^ are widely used in prostate, brain, and breast diseases [13–15]. High-b-value DWI reduces the T2 throughout effect, resulting in a higher contrast between lesions and surrounding tissues and achieving better tumor detection, especially for small lesions and abdominal hollow organ tumors [16, 17]. Background signals on high-b-value DWIs in the diagnosis of cervical cancers, intestines, and bladders showed more significantly suppressed than those in the relatively low *b* value (*b* = 800 s/mm^2^) and better efficacy of lesion detection. Decidualized endometrioma on DWIs with a *b* value of 1500 s/mm^2^ could be distinguished from ovarian cancer more easily via visual observation [18]. Although high-b-value DWI has been widely applied in clinical practices, it is challenging to obtain several high-*b*-value images for the reasons of prolonged echo time, relatively long scan time and more eddy distortion as *b* value and acquisition time increased, leading to patient discomfort, increased motion artifacts and decreased signal-to-noise ratio (SNR) [16, 19, 20]. Thus, high-b-value DWI is limited in the clinical applications.

Synthetic DWIs (syDWIs) are calculated from a group of scanned DWIs with different *b* values by extrapolating the fitted signal attenuation curve [21, 22]. SyDWIs with high *b* values showed better SNR and less image distortion than scanned high-*b*-value DWIs. They also showed a higher contrast-to-noise ratio (CNR) between the lesions and the background compared to low-*b*-value DWIs, improving the efficiency of lesion detection in research of systemic malignant tumors, prostate cancer, breast cancer, and liver metastasis [5, 23–25]. SyDWI with *b* = 1500 s/mm^2^ had higher image quality and detection rate in the diagnosis of pancreatic cancer than the real scanned DWIs with a high *b* value. Only one application of high-*b*-value syDWIs on 1.5 T demonstrated that the significance of liver metastases and detection rate were higher than the scanned ones.

Normally, two to five *b* values were set in one DWI to generate more reliable synthetic high-*b*-value DW images in the diagnosis of liver metastases [26], Crohn's disease [27], prostate [14], and pancreas [11]. In addition, the combination of small field weighted imaging and synthetic high-*b*-value diffusion-weighted imaging has been applied in pancreatic tumors [11] for improving detection efficiency. But there was no computed high-*b*-value uterus DW study to assure 5b-protocol DWI could be used in clinical diagnosis. Therefore, the feasibility and reliability of 5b-protocol DWI-generated high-*b*-value rFOV-syDWIs and rFOV-ADCs in clinical diagnosis of cervical disease should be examined using 13b-protocol DWI-generated scanned and synthetic DW images as a standard reference to compare image quality and diagnostic efficiency including lesion clarity and contrast between lesion and parenchyma.

## Materials and methods

### Patients

**Fig. 1:**
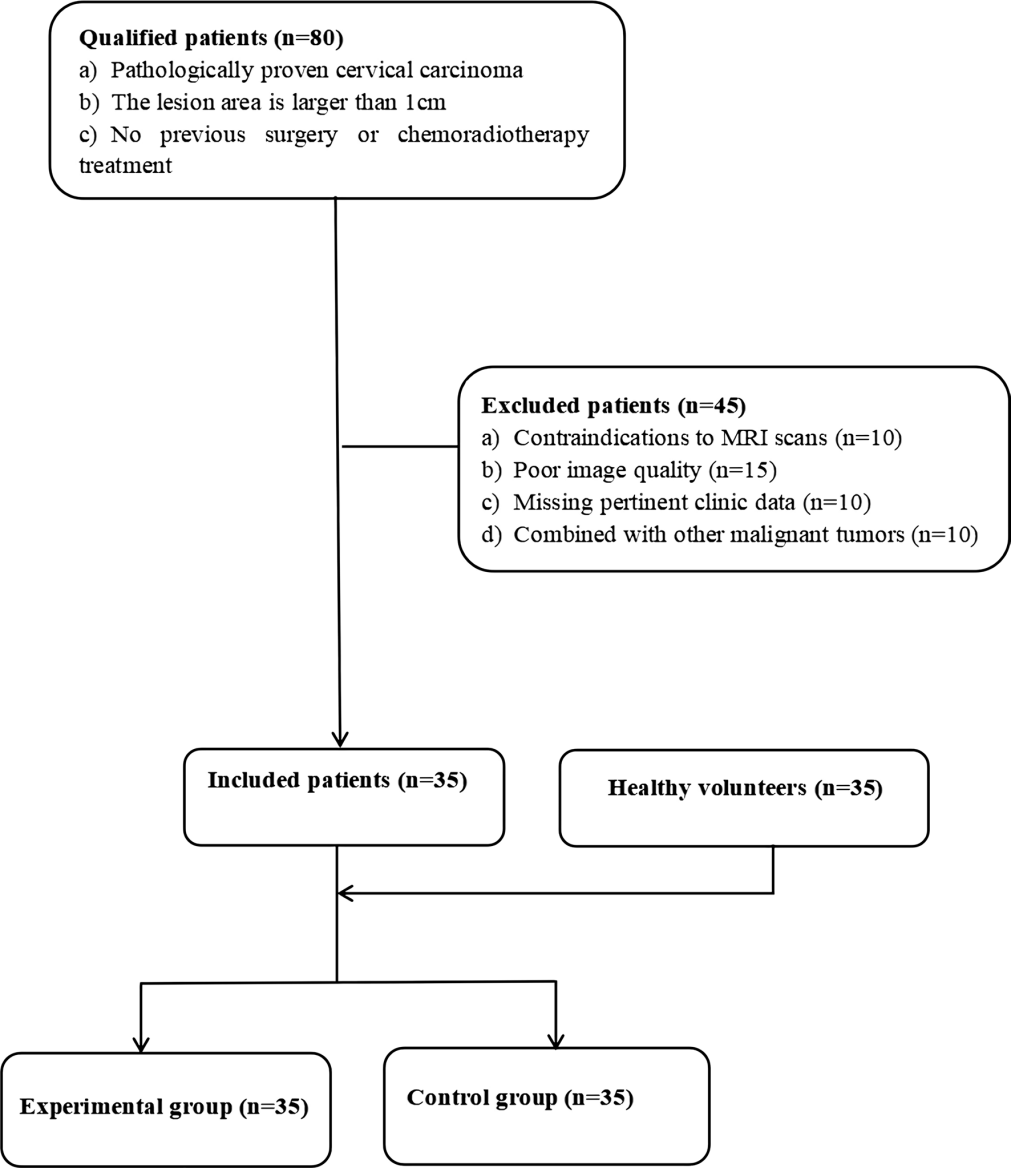
Workflow of subject inclusion and exclusion in this study

This study was approved by the institutional review board (IRB:2022KS013) of our hospital. A total of 35 patients with cervical cancer and 35 healthy volunteers who visited our hospital from May to November 2021 were prospectively recruited in this study and received clinicians’ diagnosis based on the Federation of Gynecology and Obstetrics (FIGO, 2019) [28]. All gave signed written informed consent. Inclusion criteria were as follows: (1) no contraindications to MRI scans or prior therapy; (2) no uncontrollable comorbidities or malignant tumors; (3) receiving MRI scans including a rFOV-DWI sequence; (4) good image quality without artifacts; (5) clinicopathological data were complete; and (6) the maximum diameter of the lesion was > 1 cm and the lesion can be accurately delineated (only for patients). Details are shown in Fig. 1.

### Magnetic resonance imaging and postprocessing

rFOV-DWI was acquired with TR/TE/ = 4000/71.6 ms, field of view = 20 × 20 cm^2^, matrix = 110 × 90, layer thickness = 4 mm, no gap on 1.5 Tesla (SIGNA Voyager, GE Healthcare). Two multiple-b-value protocols were used: (1) a 13b-protocol as a standard reference: *b* = 0, 25, 50, 75, 100, 150, 200, 400, 600, 800, 1000, 1200, and 1500 s/mm^2^; (2) a 5b-protocol as our proposal: *b* = 0, 100, 400, 800,1500 s/mm^2^ with NEX = 1 and 2 for *b* less and more than 200 s/mm^2^, respectively, for a total scan time of 11 min and 12 s as well as 4 min. Noteworthily, scan time depends on the number of used *b* values; meanwhile, rFOV-DWI alone elevates lesion conspicuity and edge delineation. 13b-protocol was chosen as a standard reference due to more accurate ADC maps using a mono- or bi-exponential computation model when more used *b* values are used and showing good diagnosis efficiency in cervical cancer [1, 29–32]. Each subject was instructed to properly empty the bladder to reduce artifacts of intestinal peristalsis and urine electrolyte around 30 min before scanning. All patients were free-breathing and placed in the supine position with an 8-channel phased coil over the abdomen. The top and bottom of scan coverage were positioned at the anterior and superior iliac spine and the lower margin of the symphysis pubis to include the entire uterus. rFOV-syDWIs with *b* values of 1200 (rFOV-syDWI_b=1200_) and 1500 (rFOV-syDWI_b=1500_) were automatically generated from 5b-protocol and 13b-protocol image datasets by an inline mono-exponential fitting algorithm on 1.5 T MRI console without labor consumption. Then, all 5b-protocol and 13b-protocol scanned and synthetic DW images were transferred to image postprocessing workstation (GE Advanced Workstation 4.7) and both rFOV-sADCs and rFOV-syADCs with *b* value of 1200 and 1500 s/mm^2^ were computed.

### Subjective image quality analysis

All image evaluation was performed by two radiologists with at least 10 years of experience in abdominal radiographic diagnosis. Image quality was rated on a four-point Likert scale with respect to the following four aspects (U1–U4). U1: overall image quality (4 = excellent image quality, 3 = good image quality, not affecting interpretation, 2 = fair image quality and somewhat affecting interpretation, 1 = poor image quality), U2: anatomic detail (4 = excellent delineation of anatomic structure with blurred margin, 3 = good delineation of anatomic structure with a sharp margin, 2 = fairly delineation of anatomic structure with blurred margin, 1 = poorly visualized anatomy), U3: lesion conspicuity (4 = lesions identified as distinct signal differences with a clear lesion margin, 3 = lesions identified as signal difference, 2 = lesions identified as slight signal differences, 1 = lesion not identified as signal is rough), U4: level of geometric deformation (4 = absent, 3 = mild, 2 = moderate, 1 = severe).

### Objective image quality analysis

The same two radiologists double-blindly assessed rFOV-sDWIs and rFOV-syDWIs with *b* values of 1200 and 1500 s/mm^2^. Measurements were obtained as follows: (1) average signal intensity of a lesion (${S}_{\mathrm{lesion}}$); (2) average signal intensity of gluteus maximus (${S}_{\mathrm{tissue}}$); and (3) standard deviation of signal intensity of subcutaneous fat (${\mathrm{SD}}_{\mathrm{background}}$). The following formula is used to calculate the signal-to-noise ratio (SNR) and contrast-to-noise ratio (CNR) for DWI images:

$${\text{SNR}} = \frac{{S_{{{\text{lesion}}}} }}{{{\text{SD}}_{{{\text{background}}}} }}\;\;\;{\text{CNR}} = \frac{{S_{{{\text{lesion}}}} - S_{{{\text{tissue}}}} }}{{\sqrt {S_{{{\text{lesion}}}}^{2} + {\text{SD}}_{{{\text{tissue}}}}^{2} } }}$$

### Quantitative assessment

Scanned and synthetic ADCs (sADC_b=1200/1500_, syADC_b=1200/1500_) were generated, respectively, based on rFOV-sDWIs and rFOV-syDWIs with two values of 0 and 1200 or 1500 on GE postprocessing workstation (GE Advanced Workstation 4.7). Two experienced radiologists double-blindly drew three ROIs with an area of 50 ± 5 mm on the images with the maximum lesion cross section for the patient group or maximum cervix cross-section for the healthy volunteers using T2W images as reference and placed in avoidance of visible blood vessel, tumors, hemorrhage, and necrosis. Histogram-derived parameters based on ADC values, including mean (ADC_mean_), minimum (ADC_minimum_), skewness (ADC_skewness_), and kurtosis (ADC_kurtosis_), of scanned and synthetic DWIs were then obtained.

### Statistical analysis

SPSS26.0 (IBM, Armonk, NY) was used for statistical analysis. The inter-reader differences of qualitative measurements (U1-U4) on the actual scanned DWI and syDWI were analyzed by weighted kappa analysis: 0.2 ~ 0.4 poor consistency, 0.41 ~ 0.60 medium consistency, 0.61 ~ 0.80 good consistency, > 0.81 excellent consistency. The inter-modality differences of quantitative measurements (CNR, SNR, and ADC-related values) were, respectively, examined by Wilcoxon signed-rank test or paired t test according to homoscedasticity and normality, respectively, assessed by the Levene’s test and Shapiro–Wilk test; the inter-group differences of ADC values were examined by independent t test or Mann–Whitney U test according to homoscedasticity and normality assessed by Levene’s test and Shapiro–Wilk test. All data were expressed as mean (± SD). In addition, the area under ROC curve (AUC) was compared to test the differential performance of ADCs between cervical cancer patients and healthy volunteers. *P* values < 0.05 were considered indicative of statistical significance for all tests.

## Results

### Subjective and objective image quality scores

Image quality scores of rFOV-sDWI_b=1200/1500_ and rFOV-syDWI_b=1200/1500_ computed based on 5b- and 13b-protocols were evaluated by two radiologists (Tables1 and 2). For both multiple-b-value protocols, there were statistically higher scores (U1-U4) between scanned and synthetic DWIs (*p* < 0.05) and statistically higher inter-reader agreements on synthetic DWIs (*κ* for rFOV-syDWI_b=1200_ and rFOV-syDWI_b=1500_ computed with the 13b-protocol of: U1 = 0.889, 0.886, U2 = 0.944, 0.943, U3 = 0.889, 0.886, U4 = 0.833, 0.830; with the 5b-protocol of: U1 = 0.882, 0.885, U2 = 0.935, 0.936, U3 = 0.878, 0.885, U4 = 0.833,0.830) than scanned ones (*κ* for rFOV-sDWI_b=1200_ and rFOV-sDWI_b=1500_ computed with the 13-*b*-value protocol of: U1 = 0.767, 0.759, U2 = 0.816, 0.822, U3 = 0.766, 0.771, U4 = 0.704, 0.706) (*p* < 0.001).

**Table 1:** Inter-rater agreements on subjective assessment of scanned and synthetic 13b-protocol rFOV-DWIs with *b* values of 1200 and 1500 s/mm^2^

*B* value	Overall image quality				Anatomy				Lesion conspicuity				Geometric distortion			
	Reader1	Reader2	Kappa	*p* value	Reader1	Reader2	Kappa	*p* value	Reader1	Reader2	Kappa	*p* value	Reader1	Reader2	Kappa	*p* value
s_13b_1200	1.58 ± 0.49	1.64 ± 0.48	0.767**	***p* < 0.001**	1.64 ± 0.48	1.67 ± 0.47	0.816**	***p* < 0.001**	1.61 ± 0.49	1.61 ± 0.49	0.766**	***p* < 0.001**	1.61 ± 0.49	1.64 ± 0.48	0.704**	***p* < 0.001**
sy_13b_1200	3.53 ± 0.50	3.53 ± 0.50	0.889**	***p* < 0.001**	3.53 ± 0.50	3.56 ± 0.50	0.944**	***p* < 0.001**	3.53 ± 0.50	3.53 ± 0.50	0.889**	***p* < 0.001**	3.50 ± 0.50	3.53 ± 0.50	0.833**	***p* < 0.001**
s_13b_1500	1.64 ± 0.48	1.64 ± 0.48	0.759**	***p* < 0.001**	1.60 ± 0.49	1.64 ± 0.48	0.822**	***p* < 0.001**	1.58 ± 0.48	1.58 ± 0.49	0.771**	***p* < 0.001**	1.58 ± 0.49	1.68 ± 0.47	0.706**	***p* < 0.001**
sy_13b_1500	3.58 ± 0.49	3.58 ± 0.49	0.886**	***p* < 0.001**	3.56 ± 0.50	3.59 ± 0.49	0.943**	***p* < 0.001**	3.58 ± 0.49	3.58 ± 0.49	0.886**	***p* < 0.001**	3.56 ± 0.50	3.58 ± 0.50	0.830**	***p* < 0.001**

s = scanned DWI, sy = synthetic DWI, the image scores are expressed as means ± standard deviations, *p* values < 0.001 were considered statistically significant

**p < 0.001

**Table 2:** Inter-rater agreements on subjective assessment of 13b-protocol scanned and 5b-protocol synthetic rFOV-DWIs with *b* values of 1200 and 1500 s/mm^2^

*B* value	Overall image quality				Anatomy				Lesion conspicuity				Geometric distortion			
	Reader1	Reader2	Kappa	*p* value	Reader1	Reader2	Kappa	*p* value	Reader1	Reader2	Kappa	*p* value	Reader1	Reader2	Kappa	*p* value
s_13b_1200	1.58 ± 0.49	1.64 ± 0.48	0.767**	***p* < 0.001**	1.64 ± 0.48	1.67 ± 0.47	0.816**	***p* < 0.001**	1.61 ± 0.49	1.61 ± 0.49	0.766**	***p* < 0.001**	1.61 ± 0.49	1.64 ± 0.48	0.704**	***p* < 0.001**
Sy_5b_1200	3.42 ± 0.50	3.43 ± 0.50	0.882**	***p* < 0.001**	3.51 ± 0.50	3.54 ± 0.50	0.935**	***p* < 0.001**	3.21 ± 0.50	3.21 ± 0.50	0.878**	***p* < 0.001**	3.53 ± 0.50	3.50 ± 0.50	0.833**	***p* < 0.001**
S_13b_1500	1.64 ± 0.48	1.64 ± 0.48	0.759**	***p* < 0.001**	1.60 ± 0.49	1.64 ± 0.48	0.822**	***p* < 0.001**	1.58 ± 0.48	1.58 ± 0.49	0.771**	***p* < 0.001**	1.58 ± 0.49	1.68 ± 0.47	0.706**	***p* < 0.001**
Sy_5b_1500	3.52 ± 0.49	3.54 ± 0.49	0.885**	***p* < 0.001**	3.55 ± 0.49	3.57 ± 0.50	0.936**	***p* < 0.001**	3.57 ± 0.49	3.57 ± 0.49	0.885**	***p* < 0.001**	3.50 ± 0.50	3.56 ± 0.50	0.830**	***p* < 0.001**

s = scanned DWI, sy = synthetic DWI, the image scores are expressed as means ± standard deviations, *p* values < 0.001 were considered statistically significant

**p < 0.001

**Fig. 2:**
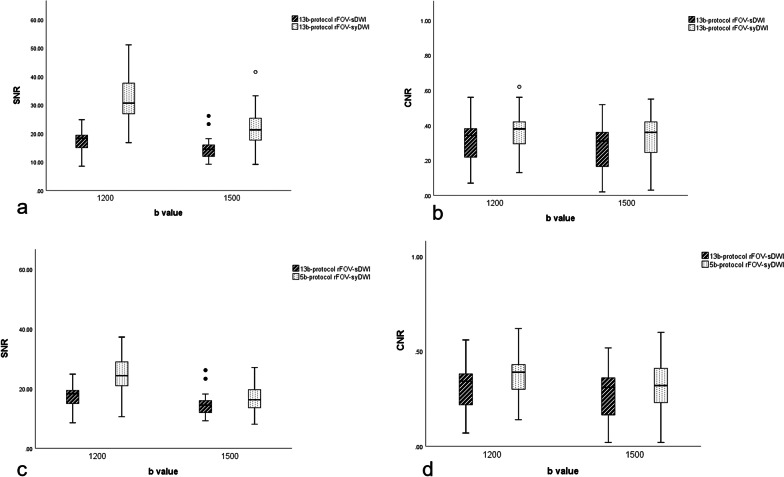
Box plots illustrating the relationship between SNR (**a**) and CNR (**b**) of 13b-protocol scanned and synthetic rFOV-DWIs. And SNR (**c**), CNR (**d**) of 13b-protocol scanned and 5b-protocol synthetic rFOV-DWIs

Objective image quality values (SNR and CNR) measured on both rFOV-sDWIs and rFOV-syDWIs with a * b* value of 1200 and 1500 are shown in Fig. 2. SNRs of 13b-protocol rFOV-syDWI_b=1200/1500_ (31.48 ± 7.44, 21.9 ± 6.01) and 5b-protocol ones (24.6 ± 6.01, 16.79 ± 4.43) were significantly higher than that of 13b-protocol rFOV-sDWI_b=1200/1500_ (17.18 ± 3.95, 14.39 ± 3.52). CNRs of 13b-protocol rFOV-syDWI_b=1200/1500_ (0.36 ± 0.11,0 0.33 ± 0.13) and 5b-protocol ones (0.36 ± 0.12, 0.31 ± 0.14) were also significantly higher than that of 13b-protocol rFOV-sDWI_b=1200/1500_ (0.32 ± 0.12, 0.27 ± 0.12).

### Quantitative assessment

**Fig. 3:**
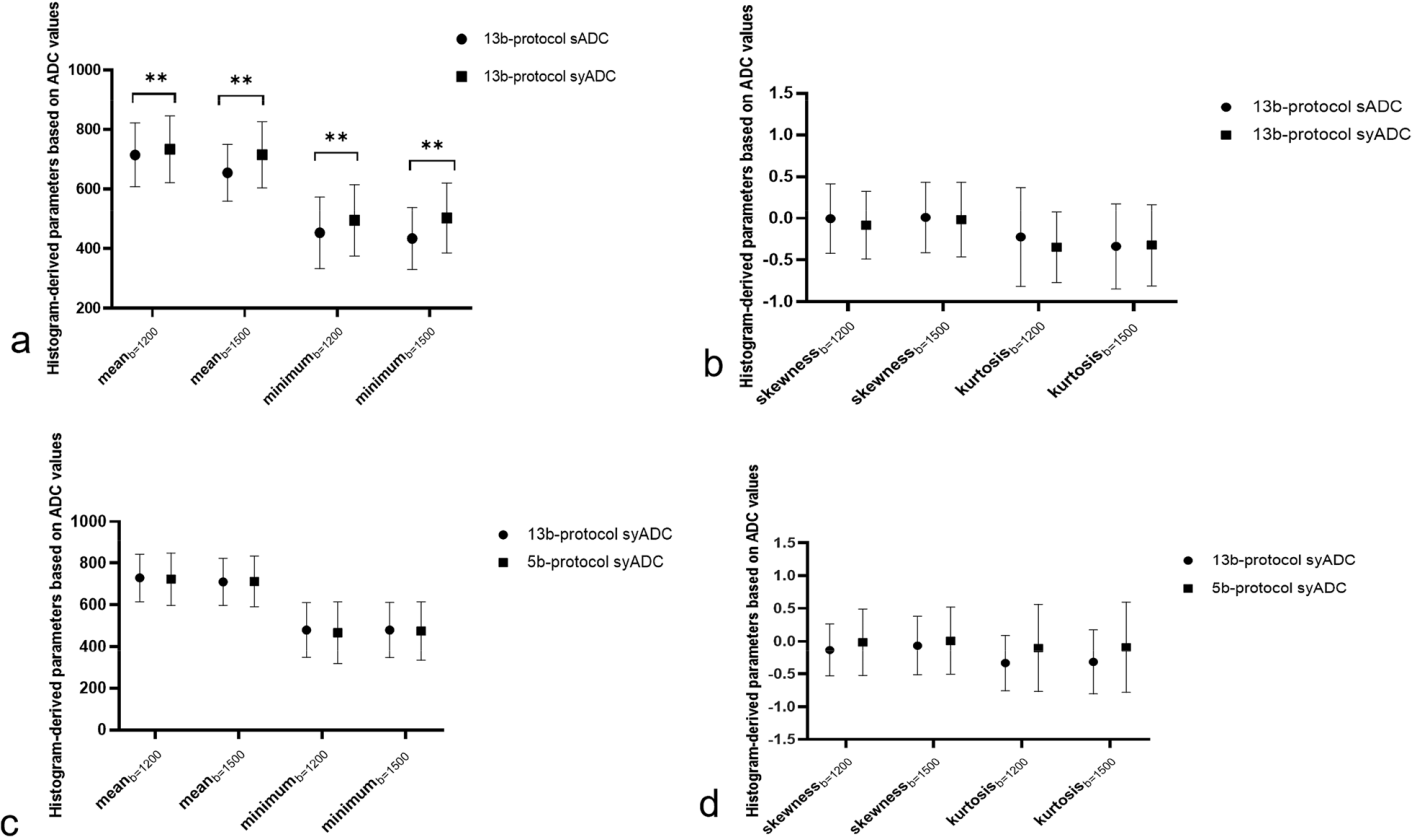
Comparisons of histogram-derived parameters (mean minimum, skewness, and kurtosis): (**a, b**) based on 13b-protocol scanned and synthetic ADC values as well as those (**c, d**) based on 5b-protocol and 13b-protocol synthetic ADC values (**p < 0.001)

Significant differences of histogram-derived ADC values in cervical cancer group were found between scanned and synthetic ADC values (Fig. 3a, b). For ADCs by 13b-protocol rFOV-syDWIs and rFOV-sDWIs, mean and minimum of syADC_b=1200/1500_ were higher than those of sADC_b=1200/1500_. For ADC computed by 5b- and 13b-protocol rFOV-syDWIs, there is no significant difference in histogram-derived parameters from both syADC_b=1200/1500_ (Fig. 3c, d).

**Fig. 4:**
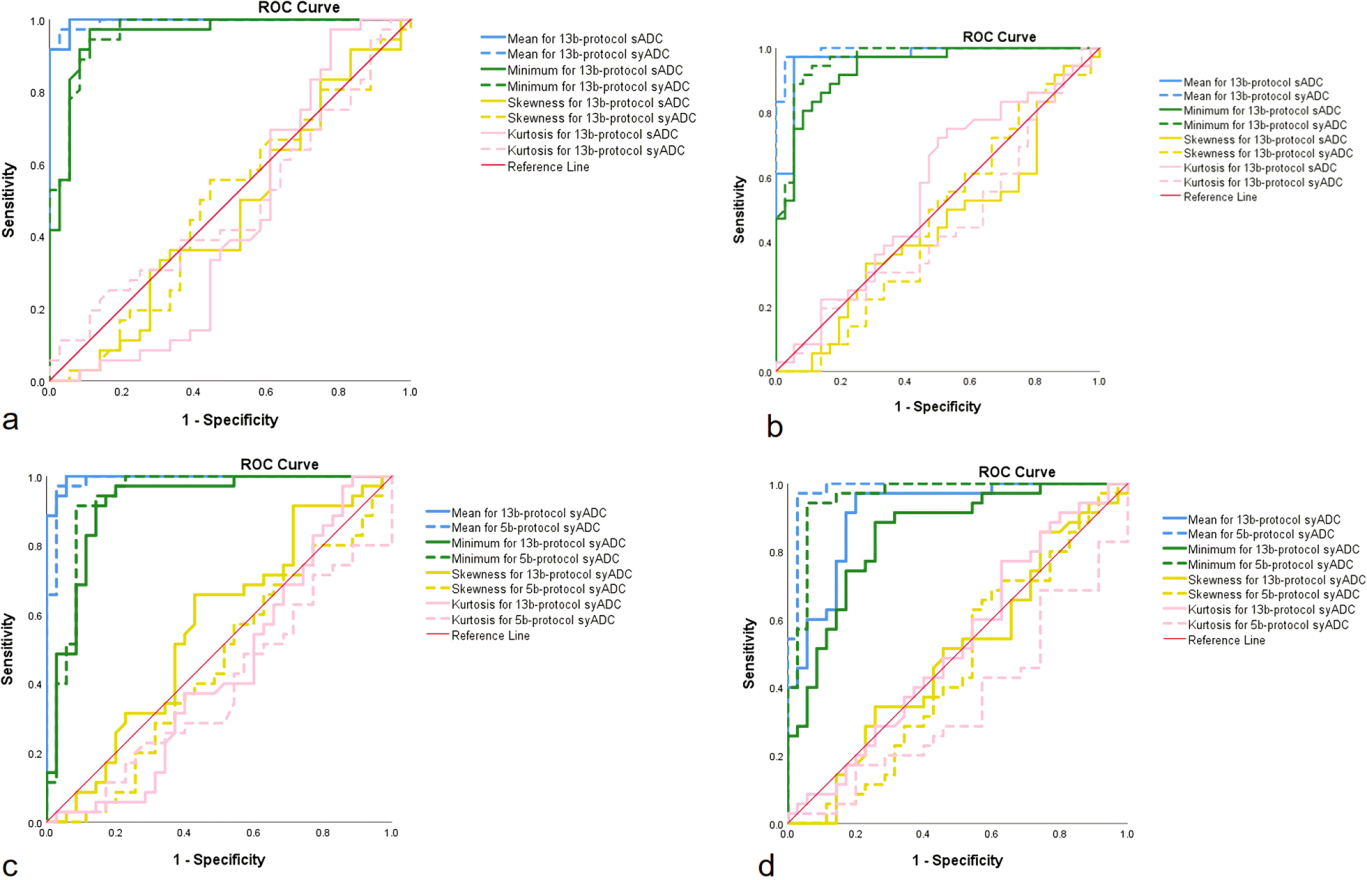
Diagnosis efficiency of scanned and synthetic 13b-protocol ADC values with a * b* value of 0 and 1200 (**a**) or 1500 (**b**) s/mm^2^ as well as that of synthetic 13b-protocol and 5b-protocol ADC values with a *b* value of 0 and 1200 (**c**) or 1500 (**d**) s/mm^2^

**Fig. 5:**
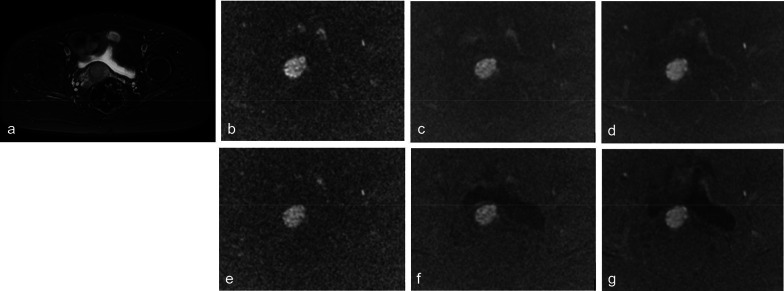
A 65-year-old woman with stage IIB cervical cancer. Illustration of the significant lesions on T2-weighted image (**a)** as well as 13b-protocol and 5b-protocol rFOV-DWI with b value of 0 and 1200 or 1500 s/mm^2^, including rFOV-sDWI_13b=1200_ (**b**), rFOV-sDWI_13b=1500_ (**e**), rFOV-syDWI_13b=1200_ (**c**), rFOV-syDWI_13b=1500_ (**f**), rFOV-syDWI_5b=1200_ (**d**), and rFOV-syDWI_5b=1500_ s/mm^2^ (**g**)

The AUCs of 13b-protocol syADC_mean_ and syADC_minimum_ were equal or higher than those of 13b-computed sADC_mean_ and sADC_minimum_ (Fig. 4a, b, Tables 3, 4). The AUCs of 5b-protocol syADC_mean_ and syADC_minimum_ were approximately equivalent to those of 13b-protocol syADC_mean_ and syADC_minimum_ (Fig. 4c, d, Tables 5, 6). The AUCs of other parameters are shown (Fig. 4, Tables 3, 4, 5, 6). Figure 5 illustrates the scanned and synthetic rFOV-DW images and a corresponding axial T2-weighted image of a patient with cervical cancer.

**Table 3:** Diagnosis performance on cervical cancer of histogram-derived scanned and synthetic 13b-protocol ADC values with a * b* value of 0 and 1200 s/mm^2^

Parameters	AUC (95%CI)	Sensitivity (%)	Specificity (%)	*p* value	Threshold (s/mm^2^)
sADC_13b,mean_	0.995 (0.987–1.000)	100.0	94.4	***p* < 0.05**	0.903 × 10^–3^
syADC_13b,mean_	0.995 (0.985–1.000)	97.2	97.2	***p* < 0.05**	0.941 × 10^–3^
sADC_13b,minimum_	0.956 (0.716–0.909)	91.7	88.9	***p* < 0.05**	0.573 × 10^–3^
syADC_13b,minimum_	0.961 (0.921–1.000)	94.4	88.9	***p* < 0.05**	0.633 × 10^–3^
sADC_13b,skewness_	0.463 (0.328–0.598)	91.7	16.7	*p* = 0.33	− 0.515
syADC_13b,skewness_	0.485 (0.349–0.620)	55.6	55.6	*p* = 0.34	− 0.067
sADC_13b,kurtosis_	0.439 (0.300–0.579)	97.2	22.2	*p* = 0.30	− 0.793
syADC_13b,kurtosis_	0.485 (0.349–0.621)	11.1	97.2	*p* = 0.35	− 0.564

*p* values < 0.05 were considered statistically significant and are shown in bolded font

AUC = area under the receiver operating characteristic curve, 95%CI = 95% confidence intervals, sADC = scanned apparent diffusion coefficient, syADC = synthetic apparent diffusion coefficient

**Table 4:** Diagnosis performance on cervical cancer of histogram-derived scanned and synthetic 13b-protocol ADC values with a * b* value of 0 and 1500 s/mm^2^

Parameters	AUC (95%CI)	Sensitivity (%)	Specificity (%)	*p* value	Threshold (s/mm^2^)
sADC_13b,mean_	0.968 (0.931–1.000)	97.2	94.4	***p* < 0.05**	0.789 × 10^–3^
syADC_13b,mean_	0.992 (0.979–1.000)	97.2	97.2	***p* < 0.05**	0.923 × 10^–3^
sADC_13b,minimum_	0.936 (0.883–0.989)	91.7	80.6	***p* < 0.05**	0.532 × 10^–3^
syADC_13b,minimum_	0.963 (0.923–1.000)	91.7	91.7	***p* < 0.05**	0.672 × 10^–3^
sADC_13b,skewness_	0.446 (0.311–0.580)	91.7	13.9	*p* = 0.31	− 0.542
syADC_13b,skewness_	0.465 (0.349–0.620)	86.1	22.2	*p* = 0.03	− 0.393
sADC_13b,kurtosis_	0.559 (0.424–0.693)	75.0	50.0	*p* = 0.42	− 0.529
syADC_13b,kurtosis_	0.451 (0.317–0.586)	100	5.6	*p* = 0.32	− 0.986

*p* values < 0.05 were considered statistically significant and are shown in bolded font

AUC = area under the receiver operating characteristic curve, 95%CI = 95% confidence intervals, sADC = scanned apparent diffusion coefficient, syADC = synthetic apparent diffusion coefficient

**Table 5:** Diagnosis performance on cervical cancer of histogram-derived synthetic 13b-protocol and 5b-protocol ADC values with a *b* value of 0 and 1200 s/mm^2^

Parameters	AUC (95%CI)	Sensitivity (%)	Specificity (%)	*p* value	Threshold(s/mm^2^)
syADC_13b,mean_	0.995 (0.985–1.000)	97.2	97.2	***p* < 0.05**	0.941 × 10^–3^
syADC_5b,mean_	0.988 (0.967–1.000)	97.1	97.1	***p* < 0.05**	0.934 × 10^–3^
syADC_13b,minimum_	0.961 (0.921–0.989)	94.4	88.9	***p* < 0.05**	0.633 × 10^–3^
syADC_5b,minimum_	0.935 (0.869–1.000)	91.4	91.4	***p* < 0.05**	0.654 × 10^–3^
syADC_13b,skewness_	0.485 (0.349–0.620)	55.6	55.6	*p* = 0.34	− 0.067
syADC_5b,skewness_	0.452 (0.316–0.588)	8.6	74.3	*p* = 0.07	− 0.239
syADC_13b,kurtosis_	0.485 (0.349–0.621)	11.1	97.2	*p* = 0.35	− 0.564
syADC_5b,kurtosis_	0.387 (0.255–0.518)	28.6	48.6	*p* = 0.07	− 0.520

*p* values < 0.05 were considered statistically significant and are shown in bolded font

AUC = area under the receiver operating characteristic curve, 95%CI = 95% confidence intervals, syADC = synthetic apparent diffusion coefficient

**Table 6:** Diagnosis performance on cervical cancer of histogram-derived synthetic 13b-protocol and 5b-protocol ADC values with a b value of 0 and 1500 s/mm^2^

Parameters	AUC (95%CI)	Sensitivity (%)	Specificity (%)	*p* value	Threshold (s/mm^2^)
syADC_13b,mean_	0.992 (0.979–1.000)	97.2	97.2	***p* < 0.05**	0.923 × 10^–3^
syADC_5b,mean_	0.984 (0.958–1.000)	97.1	97.1	***p* < 0.05**	0.910 × 10^–3^
syADC_13b,minimum_	0.963 (0.923–1.000)	91.7	91.7	***p* < 0.05**	0.672 × 10^–3^
syADC_5b,minimum_	0.962 (0.917–1.000)	94.3	94.3	***p* < 0.05**	0.645 × 10^–3^
syADC_13b,skewness_	0.465 (0.349–0.620)	86.1	22.2	*p* = 0.33	− 0.393
syADC_5b,skewness_	0.460 (0.322–0.597)	11.4	71.4	*p* = 0.07	− 0.242
syADC_13b,kurtosis_	0.451 (0.317–0.586)	100	5.6	*p* = 0.32	− 0.986
syADC_5b,kurtosis_	0.355 (0.255–0.485)	45.7	25.7	*p* = 0.07	− 0.522

*p* values < 0.05 were considered statistically significant and are shown in bolded font

AUC = area under the receiver operating characteristic curve, 95%CI = 95% confidence intervals, syADC = synthetic apparent diffusion coefficient

## Discussion

This was the first study to explore the diagnostic value of uterine tumors based on synthetic high-b-value synthetic DWIs and also ADCs using the reduced field-of-view DWI sequence. We validated 5b-protocol DWI could be an optimal approach to generate high-b-value rFOV-DW images and corresponding ADC maps due to its equivalent diagnosis performance to 13b-protocol one at both aspects of subjective and objective assessment. High-b-value rFOV-syDWIs showed better image quality (overall image quality, anatomic details, lesion conspicuity, level of geometric deformation), SNR, and CNR than rFOV-sDWIs (*p* < 0.05). Histogram-derived syADC parameters except syADC_skewness_ and syADC_kurtosis_ showed significant differences to histogram-derived sADC parameters. Both 13b- and 5b-protocol syADC_mean_ and syADC_minimum_ showed higher diagnosis performance on cervical cancer than the resting histogram-derived 13b-protocol and 5b-protocol syADC parameters and the cutoffs of syADC_mean_ and syADC_minimum_ were almost the same for both multiple-b-value acquisition protocol. All indicated that synthetic high-b-value DWIs might be an alternative image for diagnosing cervical cancers with better lesion clarity and contrast as well as possess diagnostic performance equivalent to actually scanned DWIs; however, histogram-derived synthetic ADC parameters should be cautious when used to differentiate cervical cancer from normal tissues.

The diagnostic performance of high-b-value DWI on human organs such as brain and prostate is important in spite that it may be challenging to directly acquire high-b-value images [19, 20, 33, 34]. High-b-value images often show low SNR and CNR and serious eddy current distortion due to the on–off-switched diffusion gradient and long acquisition time that might thus degrade image quality. But, scanned high-b-value DWIs of cervical cancer showed better performance on grading tumor and more signal loss in the background [35]. Therefore, the concept of synthetic high-b-value DWI was desired in clinics to overcome these drawbacks and retain the advantages. The first study of the synthetic high-b-value DWIs was explored in prostate and metastatic prostate cancer [20]. In this study, the expected signal intensity of each image voxel for high-b-value synthetic DWIs was extrapolated based on ADC maps computed using at least two low-b-value DWIs, indicating that computed high-b-value DWIs possess high SNR. In our study, four measurements in subjective evaluation of image quality (overall image quality, anatomic deformation degree, background suppression, and geometric deformation degree), two independent readers obtained higher Kappa scores on the 13b- and 5b-protocol than actual scanned DWI images with *b* = 1200 and 1500 s/mm^2^ (Tables 1, 2). In accordance with a study of ovarian cancer and prostate [18, 36], four subjective quality scores (background suppression, distortion, artifact, and overall image quality score) of sDWI_b=1500_ were higher than those of sDWI_b=1000_. Synthetic high-b-value DWIs possess improved contrast between lesions and normal tissues compared to scanned ones due to an image itself computed from low-b-value DWIs with higher SNR instead of averaging same high-b-value DWIs [37], thus slightly high CNR and increased detection rate of small lesions, pancreatic lesions, abdominal hollow organ tumors and cervical cancer [20, 21, 38]. However, scanned mammary gland DWIs demonstrated slightly higher CNR than synthetic DWIs [37] while scanned prostate DWIs showed no significant different CNR to synthetic ones. In our study, syDWI_b=1200_ and syDWI_b=1500_ showed slightly better CNR than scanned ones, partly explained for synthetic DWIs using a reduced field-of-view DWI sequence decrease magnetic inhomogeneity and dephasing-induced distortion [39]. Briefly, synthetic high-b-value DWI can highlight the tumor characteristics with higher SNR and CNR and equivalent diagnostic performance to actually scanned DWI. In addition, a combination of high-b-value DWI and T2-weighted imaging (T2WI) has been validated for improved diagnostic performance on cervical stromal invasion and the diagnostic specificity for detection of locally residual cervical tumors compared to the utility of T2WI alone [40, 41]. Therefore, rFOV-syDWIs in diagnosing cervical cancer are highly feasible in clinics.

Numerous studies have adopted ADC values in the prognosis of cervical cancer [42]; however, only some studies reported histogram-derived ADC maps in cervical cancer. An ADC value is influenced by molecular viscosity, membrane permeability, and cell structures, and histogram-derived parameters based on ADC values reflect cell heterogeneity in different physiological states. For example, the difference of ADC_mean_ values between pre- and post-concurrent chemoradiotherapy (CCRT) can predict the progression and survival of cervical cancer [43]; metastatic lymph nodes had a significantly lower ADC_minimum_ than benign lymph nodes in endometrial cancer [14]. High-grade cervical cancer also has a significantly lower ADC_minimum_ than low-grade cervical cancer [44]. Low ADC_mean_ and ADC_minimum_ were attributed that high-density tumor cells and the narrowed extracellular space limit molecular diffusion in malignant tumors [45, 46]. Similar to the previous prostate study [14, 36], both 13b- and 5b-protocol syADC_mean_ and syADC_minimum_ showed good differentiation of cervical cancer from normal tissues and equivalent or even higher diagnostic performance than sADC_mean_ and sADC_minimum_ in our study. A true high-b-value ADC can be extrapolated with relatively small errors from the low-b-value DWIs due to the log-linear relationship between ADC and *b* values [38]; accordingly, syADC_mean_ was equal to sADC_mean_ and image quality scores of rFOV-syDWIs (subjective and objective image quality) were superior to those of rFOV-sDWIs in our study as previous studies [20, 21, 38]. Although histogram-derived parameters based on synthetic ADC values (except ADC_skewness_ and ADC_kurtosis_) of both lesion and control groups were statistically different to scanned ADC values (*p* < 0.05), the ROC curves of ADC_mean_ and ADC_minimum_ but not ADC_kewness_ and ADC_kurtosis_ achieved the consistent diagnostic performance. Therefore, our study proved that syADC_mean_ and syADC_minimum_ can distinguish benign and malignant cervical lesions with extremely high diagnostic efficacy as sADC_mean_ and sADC_minimum_, and both may also play a role in prognosis such as predicting the disease progression and survival of patients with cervical cancer in the future. On the other hand, ADC_skewness_ and ADC_kurtosis_ had low diagnostic power probably due to the absence of hemorrhagic, necrotic, or cystic areas within the delineated ROIs in our study in spite that the presence of these excluding areas is considered to be an indicator of tumor heterogeneity [47]. A cervical cancer study showed ADC_skewness_ and ADC_kurtosis_ as well as the risk of lymph node metastasis elevates when the area with low ADC values (high cell density) increases [48]. A study of squamous cell carcinoma indicated that ADC_kurtosis_ reduces as a steep peak turns into a wider and flatter peak when tumors become inhomogeneous [49]. Primary tumors with higher ADC_skewness_ and ADC_kurtosis_ easily fail in chemotherapy. ADC_skewness_ and ADC_kurtosis_ are knees to increase as cell death induces tumor heterogeneity. Therefore, no effective power of ADC_skewness_ and ADC_kurtosis_ was found in our study but they still have the potential in distinguishing malignant pelvic tumors from benign ones in the future. Overall, the above-mentioned findings encouraged our team and also gave a hint to readers to observe the variance of histogram-derived ADC values in future clinical diagnosis, treatment response, and progress of cervical cancer, and assist decisions on radical hysterectomy or simple hysterectomy.

There are few limitations in our study. First, our study cohort and diversity were relatively small and this study was carried out in only one single institution. The study included patients with cervical squamous cell carcinoma but not with adenocarcinoma and small cell carcinoma. Therefore, our study results might not be generalized in all cervical diseases. Secondly, synthetic high-b-value images were conducted only on 1.5 T MRI. Direct comparison of computed DWI between different magnetic fields might be impractical and should be verified especially ADC values in disease diagnosis in spite that there is no significant difference in ADC values between cervical cancer and abdominal organs between 1.5 and 3.0 T [50]. It was worth noting that the exclusion of lesions smaller than 10 mm in our study increases the accuracy of ADC values but may lead to bias in case selection. As in previous cervical studies, the maximum cross-sectional area was used to sketch lesion ROIs and areas such as hemorrhages, necrosis, or cysts were excluded so that target ROIs showed not much heterogeneous and led to lower differentiation power. More different pathological subtypes and delineation approaches should be explored in the future to conduct a large cohort study.

In conclusion, both 5b- and 13b-protocol generated rFOV-syDWIs with better lesion contrast and higher image quality and synthetic ADC values with equivalent diagnostic power to 13b-protocol scanned ones could be applied in the diagnosis of cervical cancer, whereas synthetic ADC values should be concerned when being used to differentiation of cervical squamous cell carcinoma from benign tumors. Moreover, 5b-protocol synthetic DWIs shorten scan time and synthetic ADCs offer reliable diagnosis value for reference. Of importance, good diagnostic performance of both ADC_mean_ and ADC_minimum_ obtained using both synthetic and scanned DWIs showed reduced field-of-view DWI is reliable to be applied in clinics.

## Data Availability

The datasets generated and/or analyzed during the current study are not publicly available but are available from the corresponding author upon reasonable request.

## Abbreviations

ADC_kurtosis_: Kurtosis of the apparent diffusion coefficient
ADC_maximum_: Maximum apparent diffusion coefficient
ADC_mean_: Mean apparent diffusion coefficient
ADC_minimum_: Minimum apparent diffusion coefficient
ADC_skewness_: Skewness of the apparent diffusion coefficient
CNR: Contrast-to-noise ratio
DWI: Diffusion-weighted imaging
f-FOV: Full field of view
rFOV: Reduced field of view
rFOV-DWI: Reduced full-of-view diffusion-weighted imaging
rFOV-sDWI: Scanned reduced full-of-view diffusion-weighted imaging
rFOV-syDWI: Synthetic reduced full-of-view diffusion-weighted imaging
sADC: Scanned apparent diffusion coefficient
sDWI: Scanned diffusion-weighted imaging
SNR: Signal-to-noise ratio
syADC: Synthetic apparent diffusion coefficient
